# Molecular Imaging of Angiogenesis in Cardiac Regeneration

**DOI:** 10.1007/s12410-016-9389-6

**Published:** 2016-09-10

**Authors:** Ljubica Mandic, Denise Traxler, Alfred Gugerell, Katrin Zlabinger, Dominika Lukovic, Noemi Pavo, Georg Goliasch, Andreas Spannbauer, Johannes Winkler, Mariann Gyöngyösi

**Affiliations:** Department of Cardiology, Medical University of Vienna, Waehringer Guertel 18-20, 1090 Vienna, Austria

**Keywords:** Angiogenesis, Myocardial regeneration, Molecular imaging, Integrins, Radiotracers, Myocardial infarction

## Abstract

**Purpose of Review:**

Myocardial infarction (MI) leading to heart failure displays an important cause of death worldwide. Adequate restoration of blood flow to prevent this transition is a crucial factor to improve long-term morbidity and mortality. Novel regenerative therapies have been thoroughly investigated within the past decades.

**Recent Findings:**

Increased angiogenesis in infarcted myocardium has shown beneficial effects on the prognosis of MI; therefore, the proangiogenic capacity of currently tested treatments is of specific interest. Molecular imaging to visualize formation of new blood vessels in vivo displays a promising option to monitor proangiogenic effects of regenerative substances.

**Summary:**

Based on encouraging results in preclinical models, molecular angiogenesis imaging has recently been applied in a small set of patients. This article reviews recent literature on noninvasive in vivo molecular imaging of angiogenesis after MI as an integral part of cardiac regeneration.

## Introduction

Heart failure following myocardial infarction (MI) still displays a major cause of death and disability worldwide [1]. Even though a wide range of therapeutic options to prevent or delay transition to chronic heart failure (CHF) after MI are available, its treatment is still unsatisfactory, as CHF is generally not reversible and treatment needs to be continued indefinitely [2]. Angiogenesis, the formation of new blood vessels, is a part of the natural healing process after MI to restore blood flow and discard cellular debris [3]. The extent of angiogenesis is associated with postinfarct remodeling and has implications on prognosis in MI patients [4]. Although a variety of approaches to stimulate myocardial angiogenesis after MI have been explored, including gene therapy as well as the delivery of angiogenic factors and stem cells, results have been controversial and were partly disappointing [5–7]. In many cases, stimulation of angiogenesis was not shown convincingly and only moderate clinical improvement was demonstrated. To reliably assess the therapeutic potential of proangiogenic therapies and monitor myocardial angiogenesis for enabling better preclinical and clinical drug development, noninvasive methods such as molecular imaging are warranted. Molecular imaging of newly built microvessels is a promising strategy which allows direct visualization of vessel formation instead of indirect measurements of efficacy. Thus, it is an important modality for improving risk stratification and for facilitating the development of novel therapeutic interventions in MI patients.

### Angiogenesis

Angiogenesis represents the growth of new capillaries from preexisting vessels [8]. It is a complex process involving numerous growth factors and signal cascades [9]. Although vessels are generally quiescent in adults, endothelial cells (ECs) lining the vessel walls retain their ability to respond to angiogenic signals [8]. Proangiogenic signals such as VEGF, ANG-2, FGFs, or chemokines released by hypoxic, inflammatory, or tumor cells activate ECs, and they become motile and invasive [10]. Before ECs can sprout into surrounding tissue, degradation of basement membrane by matrix metalloproteases and detachment of mural cells is necessary in order to loosen activated ECs [8]. VEGF induces increased permeability of the EC layer, and extravasated plasma proteins serve as a provisional extracellular matrix (ECM) scaffold. Migration of ECs into this scaffold is mediated by integrins. To allow blood flow, those newly built vessels need to be connected with other vessels to build branches and become mature and stable. ECs regain their quiescent state and protease inhibitors cease basement membrane degradation [10].

Insufficient vessel maintenance can lead to MI [10]. Intact and functional blood vessels are essential for regeneration of ischemic tissues to enable immune surveillance, supply of oxygen and nutrients to and discarding of waste from the cells of the healing wound [10, 11]. Insufficiently healed MI results in an expanded infarction area and dilation of the heart by left ventricular (LV) remodeling, both resulting in heart failure [12]. However, in some patients, recovery of blood flow after MI is not possible. In those patients, restoration of tissue reperfusion depends on myocardial angiogenesis [1]. Within the first hours after MI, proangiogenic factors are released to compensate ischemia with induced angiogenesis [11]. Restoration of the blood flow in the infarct border zone is essential to alleviate infarct expansion and heart failure [1, 13]. Moreover, the extent of angiogenesis has positive effects on postinfarct remodeling and the prognosis of MI patients [4]. Hence, stimulation of myocardial angiogenesis as a therapeutic option through administering growth factors, stem or progenitor cells, and pharmacological molecules has been thoroughly studied [14]. Due to the increasing amount of research on myocardial angiogenesis as a treatment option, molecular imaging of newly built vessels has a significant potential impact on predicting outcome of MI patients and guiding novel therapies.

### Molecular Imaging Tools

Molecular imaging describes in vivo targeted, noninvasive visualization and quantification of various molecular pathways without interfering with them [15–18]. Throughout the past decades, there has been significant advances in molecular imaging techniques used for diagnostic, prognostic, as well as therapeutic purposes [18]. In the field of cardiology, molecular imaging by magnetic resonance imaging (MRI), ultrasound, bioluminescence imaging, positron emission tomography (PET), and SPECT has shown improvements of LV function, myocardial perfusion, viability, scar tissue, inflammatory cells, and indirect signs of angiogenesis, and some of these images are able to directly detect angiogenesis [15].

### Nuclear Imaging

PET imaging is a tomographic technique that detects the decay of positron emitters (radiotracers), which can be attached to small molecules for molecular recognition [17, 19]. It is well validated to have superior sensitivity, relatively high resolution, and tissue penetration [19 20•]. Various metabolic and pathophysiological biomarkers have been investigated as targets for PET imaging. The nonspecific metabolic tracer ^18^F (in form of 18-fluorodeoxyglucose), ^18^F-FDG, is the most frequently employed PET tracer [21, 22]. Many studies are directed toward incorporation of radiotracers with short half-lives, such as fluorine-18 (^18^F), which successfully leads to reduced patient exposure of ionizing radiation [23]. Rather low spatial resolution is the main limiting characteristic of this imaging technique [21].

SPECT imaging is well established and offers several advantages over PET. Camera equipment is less expensive and more widely available as compared to PET systems [23]. SPECT imaging performance is based on using single photon emitting radioisotopes, which are easier accessible for the investigation of a wider range of biological processes [15, 24]. Technetium-99m (^99m^Tc) and indium-111 (^111^In) are frequently used radioactive probes [19]. These radiotracers emit gamma rays with different energies, thus introducing the possibility of simultaneous evaluation of dual or multiple radiotracers. Advantages of SPECT are high sensitivity and tissue penetration depth. However, SPECT imaging does not ensure high-resolution anatomical information of cellular location. Another disadvantage is the inability to track radioisotopes over weeks as the signal rapidly declines [19].

### MRI

In contrast to PET and SPECT, MRI offers better spatial resolution, excellent soft tissue contrast and enables concomitant angiography or perfusion acquisition [21]. However, it has a lower sensitivity for detection of molecular contrast agents and application is limited in patients with devices, e.g., cardiac pacemakers or cardioverter defibrillators, and metal implants [15, 25]. Paramagnetic contrast agents (e.g., gadolinium) targeting integrin α_v_β_3_ via antibodies or peptidomimetics as well as gadolinium-based lipid nanoparticles, have been previously used to study tumor angiogenesis [26–28]. A further advance in MR angiogenesis imaging are ultra-small superparamagnetic particles of iron oxide (USPIO) [29]. However, USPIOs have a long blood half-life and show nonspecific extravasation [30]. Microparticles of iron oxide (MPIO) have a higher particle size, and thus a shorter half-life, offering a better contrast to noise ratio [31]. Safety concerns of superparamagnetic iron oxide particles exist [32].

### Ultrasound Molecular Imaging

Cardiac ultrasound is a widely used technique that has several advantages over the previously described imaging modalities, e.g., lack of ionizing radiation, routine accessibility, and superior spatial resolution compared to SPECT and PET [33]. Hitherto tissue perfusion assessed by ultrasound has been used as an endpoint reflecting angiogenesis; however, an increase in perfusion does not necessarily reflect angiogenic activity [34]. For more detailed imaging, targeted microbubbles can be used as contrast agents in a technique known as contrast-enhanced ultrasound (CEU) [33]. Microbubbles that target integrins or VEGFRs reflect angiogenesis in a more direct manner than perfusion imaging [34, 35].

### Bioluminescence Imaging

Bioluminescence imaging (BLI) represents an indirect cell labeling method particularly used in small animal models [36]. It is greatly valued for its high sensitivity, ease of use, and low cost of instrumentation, but BLI has low spatial resolution and restricted penetration depth, and quantification accuracy is very poor [37, 38]. Most frequently used reporter genes are firefly luciferase (Luc) and herpes simplex virus thymidine kinase (HSV-tk), used for tracking cells with angiogenic capacity [39].

### Multimodal Imaging

Advances in molecular imaging, along with identifying drawbacks, have led to the development of multimodal (hybrid) imaging systems such as PET/MR, SPECT/computed tomography (CT), and PET/CT [18]. Hybrid molecular imaging is the focus of many preclinical and clinical studies as it enables simultaneous collection of anatomical and functional information [40, 41].

The addition of CT to SPECT has permitted attenuation correction and better evaluation of SPECT myocardial perfusion [42]. SPECT/CT has proven to be relevant in the characterization of coronary artery calcium, which is a useful method to predict cardiovascular events rate [43, 44]. Even though SPECT/CT has been widely used in cardiology and information gained with this modality are highly valued, exposure of patients to radiation is a major concern [45] and reduction of radiation is the main goal of present studies in nuclear cardiology [46].

PET/CT is a hybrid technology that combines functional molecular imaging modalities with precise anatomical information [47]. This hybrid modality is successful in overcoming low spatial resolution. Many studies indicate that it results in better identification of diseases, and guide management and treatment of patients with stable and unstable coronary artery disease compared to PET imaging alone [48].

### Molecular Imaging of Myocardial Angiogenesis

Within the past decade, direct noninvasive evaluation of angiogenesis by molecular imaging has been investigated extensively. With the rapid development of antiangiogenic therapies (e.g., in cancer research) and particularly imaging techniques, tumor angiogenesis has been the focus of attention lately [49]. Although interest has recently increasingly been directed on molecular imaging of myocardial angiogenesis after MI (e.g., to monitor effects of regenerative therapies), it is still rather in its fledgling stage. Table 1 provides a summary of novel studies on molecular imaging of angiogenesis.

**Table 1:** A summary of novel studies in molecular imaging of angiogenesis

	Radiotracer	Modality	Target	Therapy	Species	Disease
Myocardial						
Preclinial	^68^Ga-NODAGA-RGD ^68^Ga-TRAP(RGD)_3_ ^18^F-galacto-RGD [50]	PET	α_v_β_3_ integrin	None	Rat	MI
	^68^Ga-NOTA-RGD peptidomimetic [51]	PET	α_v_β_3_ integrin	None	Rat	MI
	^18^F-Alfatide II [20•]	PET	α_v_β_3_ integrin	VEGF, BMSC	Rat	MI
	^68^Ga-RGD [52•]	PET	α_v_β_3_ integrin	Dissociated HUVECs/cbMSCs or 3D HUVEC/cbMSC aggregates	Rat	MI
	^11^In-DTPA-cNGR [53]	SPECT	CD13	None	Mouse	MI
	^64^Cu-NOTA-TRC105 [54]	PET	CD105	None	Rat	MI
	[^11^C]ATV-1[9]	PET	VEGFR-2, Tie-2, PDGFα	None	Rat	MI
Clinical	^68^Ga-PRGD2 [55••]	PET	α_v_β_3_ integrin	None	Human	MI
Hind limb						
Preclinical	α_v_ targeted microbubbles [34]	Ultrasound	α_v_ integrins	HIF-1α mutants	Mouse	Ischemic hind limb
Tumor						
	c(RGDyK)-MPIO [56•]	MRI	α_v_β_3_ integrin	None	Mouse	Melanoma, colon carcinoma
	^68^Ga-aquibeprin [57••]	PET	α_5_β_1_ integrin	None	Mouse	melanoma

Angiogenesis as a multistep process, orchestrated by a wide range of growth factors, growth factor receptors, cell types, adhesion molecules, integrins, and signaling pathways, all of which offer a multitude of imaging targets. In general, three ways to image myocardial angiogenesis exist: (1) non-EC targets, (2) EC targets, and (3) extracellular matrix proteins and matrix proteases [58]. In particular, integrin α_v_β_3_ has emerged as an interesting target.

### Integrins

Integrins are structurally and functionally diverse families of cell adhesion molecules, which regulate cell-cell and cell-ECM interactions and in addition mediate signals for cell growth, proliferation, migration, or apoptosis [59]. They connect the ECM with the cytoskeleton (i.e., the microfilaments) inside the cell and transmit signals of the surrounding into the cell by mediating the downstream consequences of cell adhesion. Therefore, integrins play an important role in cell signaling and can have a relation to cell growth, cell division, cell survival, differentiation, and apoptosis [60]. Several members of the integrin family are overexpressed on ECs under hypoxia [61]. The two main integrins α_v_β_3_ and α_5_β_1_ facilitate several mechanisms during angiogenesis in tissue ischemia. In particular, they mediate adhesion to ECM and other cells to initiate building of new capillaries by allowing ECs to bind to provisional ECM scaffold proteins. Furthermore, they mediate interaction of ECs and vascular smooth muscle cells, stimulate vessel growth, and promote vessel maturation [10, 61]. ECM proteins such as fibronectin interact with integrins via the Arg-Gly-Asp (RGD) sequence motif [61]. Multivalent binding is mediated through extracellular integrin clusters, and thus, dimeric and multimeric RGD sequences with improved binding affinity have been developed and are frequently used for integrin imaging.

### Integrin α_v_β_3_

Integrin α_v_β_3_-mediated imaging is currently the most frequently applied method to visualize angiogenesis in vivo. Its expression is low in normal tissue, but it becomes highly expressed in activated ECs during angiogenesis in the infarcted myocardium [61, 62]. However, studies in α_v_- and β_3_-deficient mice suggest that both integrins are not essentially required for angiogenesis and their absence can be compensated by upregulation of VEGFR-2 expression [63–65]. Additionally, integrin α_v_β_3_ does not seem to be restricted to ECs but is also expressed on macrophages so that results in angiogenesis imaging targeting integrin α_v_β_3_ need to be treated with caution [66].

**Fig. 1:**
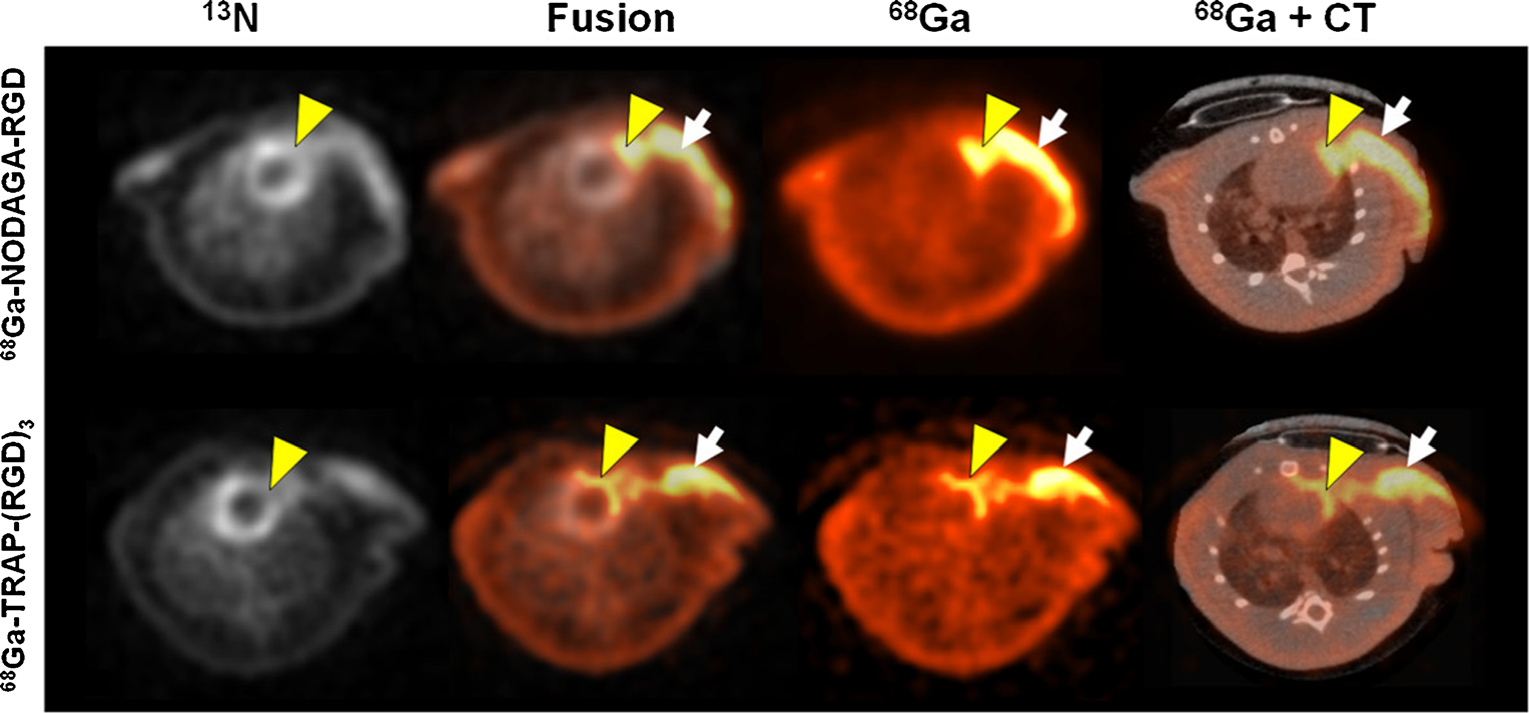
In vivo PET/CT images of rat MI. Representative transaxial sections show hypoperfused myocardium area (^13^N) and corresponding RGD uptake (^68^Ga, % ID/g). The focal uptake is seen in infarct (*yellow arrowheads*) and the operation scar (*white arrows*), as verified by CT scan (reprinted from [50] under the terms of the Creative Commons Attribution License 2.0)

**Fig. 2:**
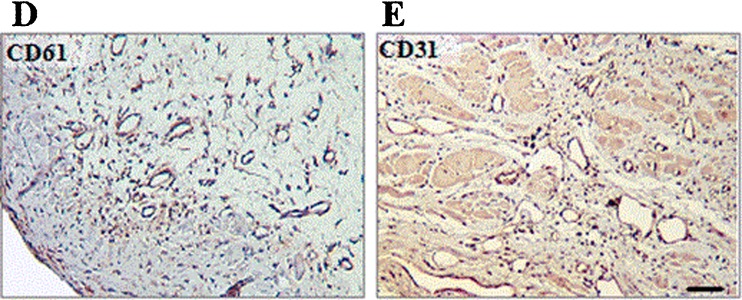
Macrosections and microsections of rat hearts. […] **d**, **e** Representative immunostaining for CD61 (**d**) and CD31 (**e**) from the border region of infarcted heart at low magnification (×10, calibration bar 50 μm) (reprinted from [51] with permission from Springer/European Journal of Nuclear Medicine)

For integrin α_v_β_3_ molecular imaging, cyclic RGD dimers with polyethylene glycol spacers radiolabeled with ^18^F [67, 68], ^68^Ga [69, 70], ^64^Cu [71], ^76^Br [72], and ^89^Zr [73] for PET imaging and ^99m^Tc [74, 75] and ^111^In [76] for SPECT imaging were used in several disease entities. Within the past years, the use of these and other tracer probes that had been investigated predominantly in tumor angiogenesis has been translated to angiogenesis imaging after MI for evaluating proangiogenic effects of regenerative therapies. In a previous study, ^18^F-galacto-RGD injected in a rat MI model predicted improved healing [77]. The usage of this tracer, however, might be limited as the production of ^18^F-galacto-RGD is complex and time-consuming. ^68^Ga tracers, on the other hand, are easy to handle and fast in production. ^68^Ga-NODAGA-RGD and ^68^Ga-TRAP-(RGD)_3_ have been previously tested for angiogenesis imaging in tumor models [78, 79]. Both ^68^Ga-RGD tracers were compared to ^18^F-galacto-RGD in postinfarct myocardial angiogenesis, and uptake was similar in all three groups (Fig. 1), indicating that ^68^Ga-RGD tracers may represent a more easily clinically translatable alternative [50]. Another ^68^Ga-labeled tracer, a ^68^Ga-NOTA-RGD peptidomimetic, was used for angiogenesis imaging in a rat MI model. ^68^Ga-NOTA-RGD uptake was increased in regions of reduced myocardial perfusion and correlated with immunohistochemical staining of CD31 and β_3_ integrin (Fig. 2) [51].

**Fig. 3:**
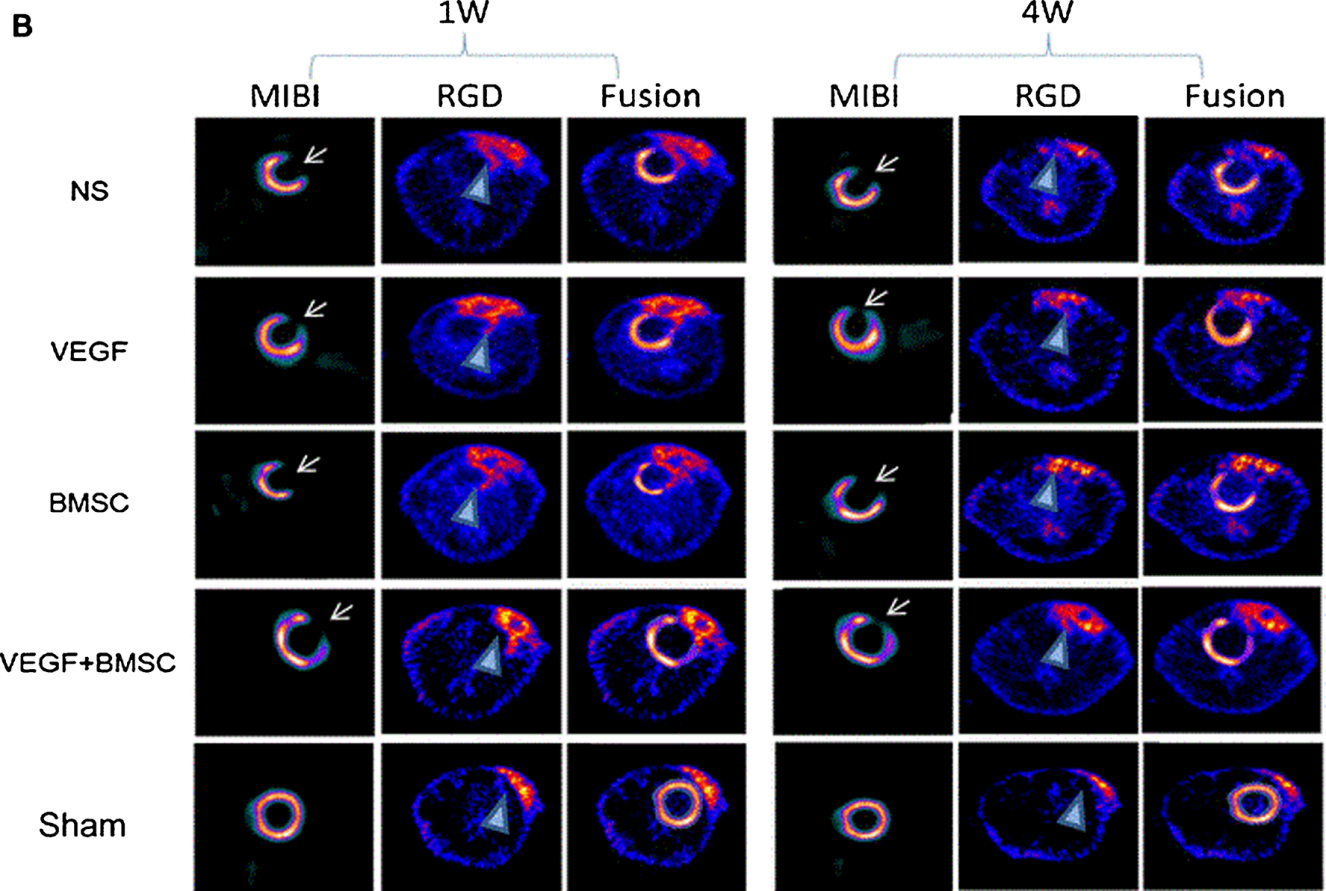
[…] **b** In vivo PET images of 18F-Alfatide II and representative SPECT myocardial short axis slice images using 99mTc-MIBI at different times after myocardial infarction. Infarcted myocardium showed obvious 99mTc-MIBI uptake defect in the anterior and lateral wall of left ventricle (*arrows*), which matched the focal RGD peptide tracer uptake region (*triangle*). **c** The infarct area/remote area ratio of 18F-Alfatide II uptake as measured by PET (reprinted from [20•] with permission from Springer)

Lately, ^18^F-Alfatide II (^18^F-AlF-NOTA-PRGD2) has been developed as a new promising PET tracer. Taking advantage of the preformation of an aluminum-fluoride complex with consequent attachment of the RGD peptide, time for preparation was significantly reduced and HPLC purification was avoided, while receiving radiochemical purity of over 97 % [80]. The ^18^F-Alfatide II tracer was used to characterize angiogenesis in a rat MI model after treatment with vascular endothelial growth factor (VEGF) gene and/or bone marrow mesenchymal stem cells (BMSCs). In this study, ^18^F-Alfatide II provided a strong contrast between infarcted and noninfarcted myocardium and uptake was significantly higher in rats treated with VEGF and BMSCs (Fig. 3). Increased uptake of ^18^F-Alfatide II correlated with the area of ^99m^Tc-MIBI uptake defect [20•].

**Fig. 4:**
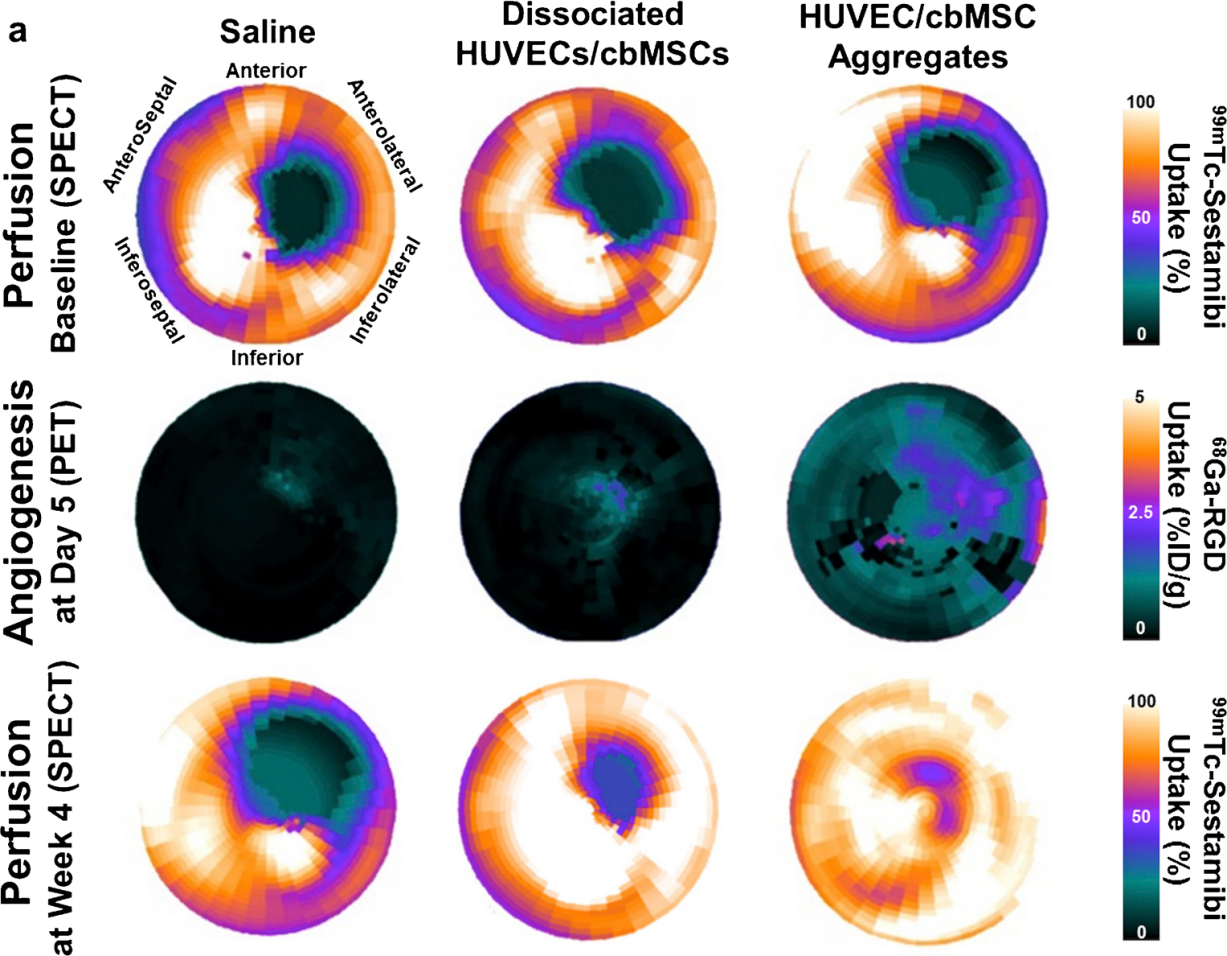
Multimodality noninvasive imaging by SPECT and PET, showing myocardial perfusion and angiogenesis, respectively. **a** SPECT and PET images in polar-map format, showing perfusion defects and angiogenesis of infarcted hearts that were treated with saline, dissociated cells, or cell aggregates. […] (reprinted from [52•] with permission from Elsevier)

In another study in a rat MI model, the angiogenic potential of 3D HUVEC/cbMSC aggregates was assessed by ^68^Ga-RGD. Injection of 3D HUVEC/cbMSC aggregates resulted in locally increased ^68^Ga-RGD uptake suggesting increased angiogenesis and reduction in defect size [52•]. Using SPECT tracers, an increased uptake of ^99m^Tc-labeled RGD peptides (^99m^Tc-RAFT-RGD and 99mTc-NC100692), similar to data of respective ^18^F-PET tracers, was found in infarcted myocardium tissue and the border zone of infarction, indicating increased integrin α_v_β_3_ expression (Fig. 4) [52•, 81, 82].

**Fig. 5:**
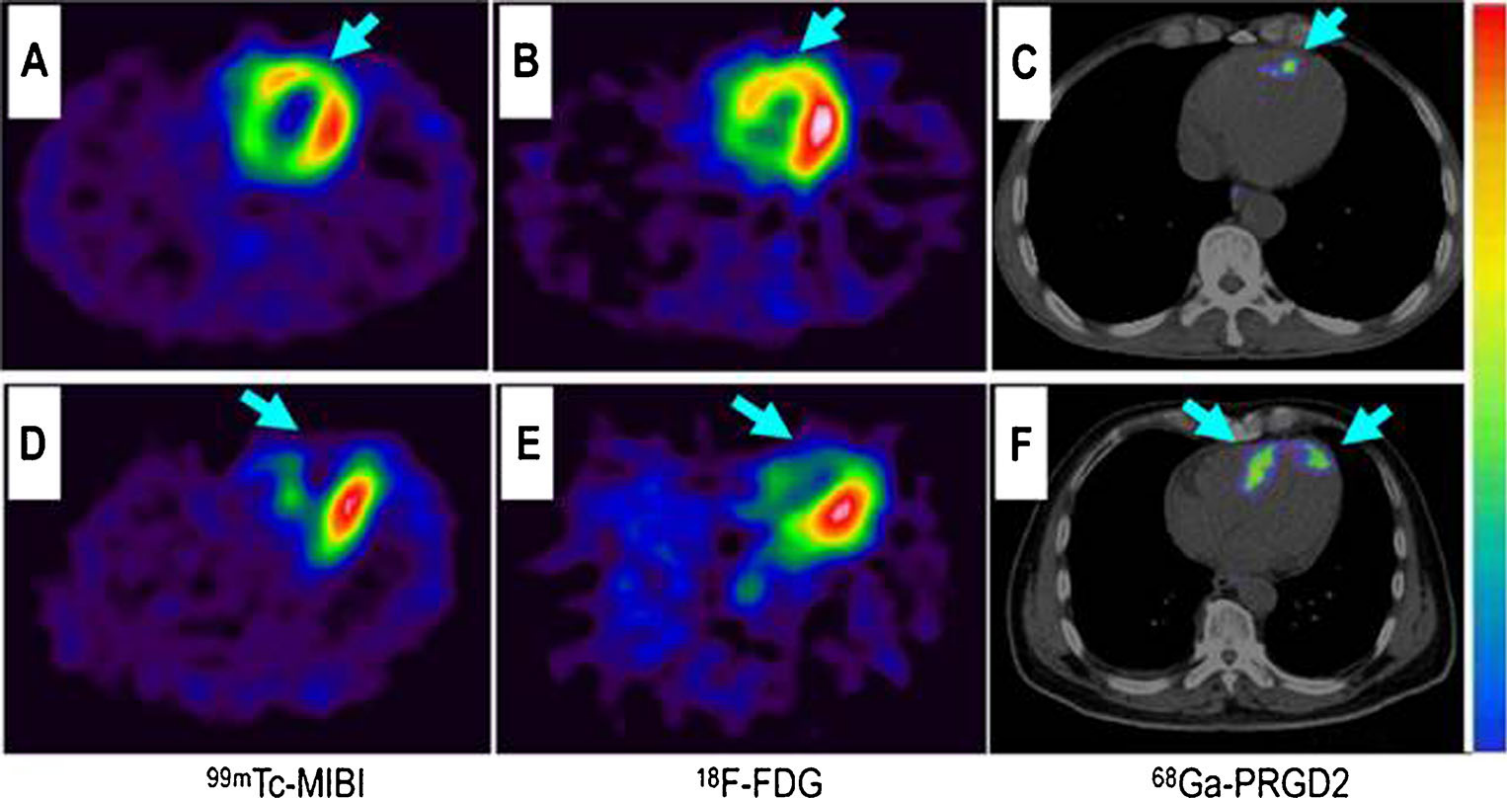
Comparison of a patient with slight myocardial infarction (MI) and a patient with severe MI. *Upper row*: In a 58-year-old man at the fifth day after the event, a small apical region with decreased 99mTc-MIBI perfusion (**a**, *arrow*) and 18F-FDG metabolism (**b**, *arrow*) showed mild 68Ga-PRGD2 accumulation (**c**, *arrow*), with a pSUV of 0.62. *Lower row*: In a 45-year-old woman on the seventh day after the event, an apical defect on 99mTc-MIBI perfusion images (**d**, *arrow*) and 18F-FDG metabolism images (**e**, *arrow*) corresponded with moderate 68Ga-PRGD2 uptake (F, *arrows*), with a pSUV of 2.02 (reprinted from [55••] with permission from Theranostics)

Recently, a ^68^Ga-labeled cyclic RGD dimer with a PEG spacer (^68^Ga-PRGD2) was studied for the first time in a small set of patients post-MI. ^68^Ga-PRGD2 uptake was found in 20 of 23 patients around the ischemic regions. Increased uptake was found 1 week after MI and remained high until 2.5 months after MI. ^68^Ga-PRGD2 uptake correlated with size and severity of the infarction (Fig. 5). Three patients who did not show any ^68^Ga-PRGD2 uptake were identified with a very recent MI and events dated back 1–2 years. Nevertheless, ^68^Ga-PRGD2 uptake showed a patchy pattern, which may be attributable to an uptake not only by angiogenic ECs but also interstitial myofibroblasts contributing to myocardial remodeling [55••]. Although the application of integrin α_v_β_3_ imaging has been previously translated into clinical trials to assess tumor angiogenesis [83], only a single clinical trial imaging angiogenesis after MI has been conducted so far, with two studies currently recruiting (NCT01813045, NCT01542073).

Even though recent work in integrin α_v_β_3_ imaging with PET or SPECT registered substantial improvements, this method is still afflicted with several limitations. Recently, a cyclic RGD moiety conjugated to MPIO (c(RGDyK)-MPIO) for MR angiogenesis visualization in a colorectal carcinoma and melanoma mouse model was studied. c(RGDyK)-MPIO specifically binds to integrin α_v_β_3_ expressing vessels, while unbound particles are rapidly cleared from circulation. Specific binding was verified by ex vivo immunolabeling [56•]. Further optimization of MR-based angiogenesis imaging tracers may enable integrated molecular and anatomical imaging.

CEU imaging with targeted microbubbles displays another radiation-free molecular imaging technique. Microbubbles binding to integrin α_v_β_3_ and other angiogenesis specific targets have been extensively applied for studying angiogenesis and the response to antiangiogenic therapies in several tumor entities [35, 84–86]. However, only limited data exist regarding cardiovascular diseases and treatments [87]. Xie et al. investigated the effect of HIF-1α mutant on angiogenesis in a mouse ischemic hind limb model; CEU imaging using α_v_-integrin-coated microbubbles has been applied to visualize angiogenesis. Video intensity obtained by α_v_ imaging positively correlated with ultrasound perfusion imaging data, indicating that CEU imaging may also provide quantitative data on angiogenesis. This suggests that α_v_-integrin imaging via ultrasound can be a reliable method to visualize angiogenesis in vivo [34]. Moreover, ultrasound is not only limited to imaging angiogenesis to monitor cardiac regeneration but offers therapeutic options. Microbubbles loaded with therapeutic agents can be dissolved by high acoustic pressures after accumulation at the region of interest, thus enabling targeted drug delivery. Additionally, it is hypothesized that a combination of targeted imaging and drug release via microbubbles is possible [33, 88, 89].

### Integrin α_5_β_1_

Integrin α_5_β_1_ expression is suggested to be completely restricted to ECs as deletion of the β_1_ chain leads to full inhibition of angiogenesis [90]. Analogous to integrin α_v_β_3_, expression of integrin α_5_β_1_ is low in quiescent ECs and upregulated in angiogenic ECs [91, 92]. These results suggest that integrin α_5_β_1_ could be a more reliable biomarker for angiogenesis compared to α_v_β_3_. To assess integrin α_5_β_1_ imaging in tumor angiogenesis, Notni et al. developed ^68^Ga-aquibeprin (a pseudopeptide targeting integrin α_5_β_1_) and compared it to ^68^Ga-avebetrin (targeting integrin α_v_β_3_). In vitro data showed high affinity for integrin α_5_β_1_, and no decrease in specificity compared to a previously used ^68^Ga-labeled monomer selectively targeting integrin α_5_β_1_ was detected. In vivo data showed a higher tumor-to-organ ratio of ^68^Ga-aquibeprin and suggest it to be sufficiently stable. Immunohistochemical stainings further propose integrin α_5_β_1_ as a more EC specific marker [57••].

### Other targets for molecular imaging

#### CD13

CD13 is a membrane-bound aminopeptidase, which is upregulated on activated ECs [93]. It is considered an important regulator of EC morphogenesis during angiogenesis [94]. The cyclic tripeptide Asn-Gly-Arg (cNGR) binds to CD13 on activated ECs in infarcted myocardium, but not to CD13-positive macrophages in hypoxic myocardium [53, 95]. Comparative studies with RGD and NGR of tumor angiogenesis revealed a threefold higher target homing ratio for NGR [96]. A recent study investigated CD13-targeted angiogenesis imaging in a mouse model of MI with ^111^In-DTPA-cNGR by SPECT. Increased uptake of ^111^In-DTPA-cNGR at day 7 after MI correlated with areas of decreased ^99m^Tc-sestamibi [53].

#### CD105

CD105 (endoglin) is a transmembrane protein that is solely expressed on activated ECs [97]. Several PET probes based on TRC105, a monoclonal antibody that binds to CD105 with high avidity, have been tested for tumor angiogenesis imaging [98, 99]. ^64^Cu-NOTA-TRC105 was recently tested to assess angiogenesis in a rat MI model via PET. Tracer uptake was increased in infarcted myocardium. Expression of CD105 was confirmed by immunofluorescence. However, ^64^Cu-NOTA-TRC105 exhibits a long half-life and its intense background signal acted as a confounder [54].

#### VEGF

VEGF is commonly considered as the most potent mediator of angiogenesis. Consequently, VEGF and its receptors (VEGFRs) are frequently used for angiogenesis imaging. Due to splice variants, several isoforms of VEGF-A exist, of which some are proangiogenic and other antiangiogenic [100]. Monoclonal human anti-VEGF labeled with ^123^I and ^124^I have been employed for PET/SPECT imaging [101]. In a rat model of myocardial infarction, recombinant radiolabeled VEGF (^64^Cu-DOTA-VEGF_121_) was used for PET imaging of VEGFRs. An increased radiotracer uptake was reported in a period of up to 2 weeks after induction of MI [101].

Several tyrosine kinases are upregulated in heart tissue undergoing angiogenesis and remodeling after MI. Immunohistochemical analyses of MI samples revealed increased levels of VEGFR-2, Tie-2, and PDGFα suggesting its use as an angiogenesis marker in non-invasive molecular imaging. ATV-1 can act as an inhibitor of those kinases. PET imaging with [^11^C]ATV-1 was assessed in a rat model of MI. Standard uptake values of [^11^C]ATV-1 correlated with immunohistochemical staining of VEGFR-2, Tie-2, and PDGFα [9].

## Conclusions

Even though proangiogenic therapies have so far largely failed as an effective treatment of MI, targeting angiogenesis after MI to mitigate heart failure is still considered a promising strategy. In order to assess the success of such therapeutic interventions in the clinic or in preclinical development, reliable and sensitive noninvasive imaging modalities are needed. Molecular imaging of angiogenesis via PET, SPECT, MRI, and CEU has been investigated intensively within the past decade. A variety of tracers have been translated from tumor angiogenesis models to MI models, and promising results were achieved. These methods offer the unique opportunity to study in vivo molecular mechanisms characterizing myocardial healing after infarction and to evaluate angiogenic effects of regenerative treatments. Combination of high sensitivity PET and SPECT with high-resolution X-ray CT images allows better identification and quantification of tracer uptake within the region of interest. Multimodal imaging with highly specific tracers yields reliable and detailed data of cardiac angiogenesis in small and large animal models and also in humans. Because PET and SPECT imaging use ionizing radiation, these imaging modalities might also expose patients to a risk of growing neoplastic lesions. For clinical applications, further research is warranted to develop radiotracers with a reasonable level of ionizing radiation or even replacing PET and SPECT with MRI or CEU while still featuring high affinity for visualizing growing blood vessels. Continuing development of noninvasive imaging modalities for future clinical applications may enable improved patient risk stratification and pave the way for personalized therapy.
